# Work Stress, Premenstrual Syndrome and Dysphoric Disorder: Are There Any Associations?

**Published:** 2011-03-01

**Authors:** B Namavar Jahromi, S Pakmehr, H Hagh-Shenas

**Affiliations:** 1 Department of Obstetrics and Gynecology, Shiraz University of Medical Sciences, Shiraz, Iran; 2 Psychiatry Research Center, Shiraz University of Medical Sciences, Shiraz, Iran

**Keywords:** Premenstrual syndrome, Premenstrual dysphoric disorder, Work stress, Female, Medical students

## Abstract

**Background:**

Women with recurrent and severe symptoms are diagnosed as having premenstrual syndrome (PMS), and if they suffer from severe affective symptoms, a diagnosis of premenstrual dysphoric disorder (PMDD) is made. The purpose of this study was to determine the association of work stress with PMS and PMDD.

**Methods:**

Fifty-five female medical students in their internship program (ten 24-hour shifts per month) and 38 third-year female medical students without any shift duties were asked to participate in this study. A questionnaire was used to record demographic information and a self-report inventory was used to measure 13 symptoms relevant to PMS and PMDD according to DSM-IV criteria. All participants were asked to complete the inventory every night around midnight for those on shifts or before going to bed at home for 60 consecutive nights.

**Results:**

Out of 55 volunteers in the shift-work group, 31 (56%) fulfilled the diagnostic criteria for PMS in contrast to 12 (32%) in the control group. The frequency of PMDD was 12 (22%) in the intern group and 5 (13%) in the control group. Twenty one students (55%) from the control group did not have PMS or PMDD, compared to 12 (22%) students from the shift workers. Decreased energy (70.9%) and irritability (65.4%) were the most frequent symptoms during the luteal phase in the shift-work group.

**Conclusion:**

Work stress and an increase in responsibility may produce or exacerbate PMS. Self-help approaches to induce self-awareness, along with psychological and psychiatric interventions, may help susceptible women to overcome this cyclic condition in order to increase their productivity as well as their quality of life.

## Introduction

Premenstrual syndromes, a collection of somatic and psychological symptoms recurring specifically during the luteal phase of each cycle and resolving by the end of menstruation, are prevalent among women of reproductive age.[1] These symptoms can range from a minor inconvenience to severe debilitation with impairment of normal functioning in relationships, work and social activities. Women with recurrent and more severe symptoms are diagnosed as having premenstrual syndrome (PMS), and if they suffer from severe affective symptoms, a diagnosis of premenstrual dysphoric disorder (PMDD) is made.[2]

Few studies have indicated that work-related stress may increase PMS.[3][4] The perception of having more work pressure, less autonomy on the job and less variety in the work conditions has been shown to be significantly higher among women with PMS in contrast to those without symptoms.[5] This study was designed to evaluate the effect of shift-work stress and direct patient responsibilities on PMS and PMDD in a cohort of female medical students. The incidence of PMS and PMDD were compared between medical students with 24-hour shift work (10 shifts per month) and those without this responsibility.

## Materials and Methods

A total of 124 single female medical students of Shiraz University of Medical Sciences participated in this study. Seventy-seven cases with internship program formed the study group who had a strictlyscheduled training program with 10 clinical shifts per month. The control group consisted of 47 third-year female medical students who did not have shift work responsibilities. Interns in the shift-work group had bedside duties 6 days a week in addition to ten 24-hour shifts monthly with direct patient responsibility. The third-year students had to attend at least 6 hrs of formal classes, 6 days a week but had no night duties or hospital work except few hours of bedside observing programs.

The participants were asked to complete 2 questionnaires. A especially-designed demographic questionnaire included items relevant to personal, menstrual, medical and drug history, which was completed at the beginning of the study. Also, a self-report inventory based on DSM-IV (American Psychiatric Association 1994)[6] was used to identify criteria for somatic and affective changes in the menstrual cycle. Thirteen items in the inventory asked about the presence of symptoms of PMS and PMDD and their effect on daily functioning. The participants were instructed to note their symptoms every night around midnight for those on shifts or before going to bed at home.

The luteal phase was defined as the 14 days prior to the first day of the next menstruation. PMS was diagnosed if the symptoms occurred in the luteal phases of two cycles and if the symptoms were severe enough to disrupt relationships, work or social activities. A diagnosis of PMDD was made if the subjects reported five or more symptoms, including at least one dysphoric symptom (irritability, anxiety, liability or depressed mood), severe enough to interfere with normal activities.[7]

Exclusion criteria were using oral contraceptives or other hormonal medications, irregular menses and documented organic or psychiatric disorders. This project was approved by the Ethics Committee of Shiraz University of Medical Sciences. Statistical analyses were done by SPSS software (Version 15, Chicago, IL, USA) using Chi-Square and t tests. Statistical significance was set at p<0.05.

## Results

After applying all exclusion criteria, 55 participants from the 24-hour shift-work group and 38 women from the control group were enrolled. The mean age for the case group was 24.33±1.3 years and 22.03±0.75 years for the control group ranging from 20 to 30 years old. Table 1 summarizes the demographic characteristics of the subjects.

**Table 1:** Demographic characteristics of shift group (case) and non-shift group (control).

****	**Case (No.=55)^a^**	**Control (No.=38)^a^**	**95%CI for mean differences**	**P value**
Mean age of menarche (years)	13.55±1.31	13.61±1.07	(-2.7, -1.8)	0.81
Mean cycle length (days)	28.42±1.57	27.5±2.21	(-1.75, -.08)	0.03
Mean duration of menstruation (days)	6.05±1.38	5.82±1.22	(-0.79, 0.31)	0.39

^a^ The values are expressed as mean±SD

The frequency of each premenstrual symptom in shift workers and the control group is presented in Table 2. All somatic and behavioral symptoms were significantly more frequent in the shift-work group. Decreased energy or fatigue (70.9%) was the symptom reported most frequently. From the affective symptoms, anger and/or irritability was significantly more frequently reported in the case group (p<0.05).

**Table 2:** Comparison of symptoms between shift workers and the control group

**Symptoms**	**Case(No.=55) No.(%)**	**Control(No.=38) No.(%)**	**Chi-Square**	**p value**
Abdominal cramps	31 (56.4)	11 (28.9)	6.82	0.009
Joint or muscle pain	23 (41.8)	8 (21)	4.36	0.037
Bloating or weight gain	21 (38.2)	6 (15.8)	5.46	0.019
Breast tenderness	24 (43.6)	6 (15.7)	7.97	0.005
Change in appetite	20 (36.4)	5 (13.2)	6.15	0.013
Change in sleep pattern	25 (45.4)	6 (15.7)	8.90	0.003
Difficulty in concentrating	19 (34.5)	4 (10.5)	6.96	0.008
Decreased energy (fatigue)	39 (70.9)	14 (36.8)	10.64	0.001
Decreased interest in activity	33 (60)	13 (34.2)	5.97	0.014
Mood swing	32 (58.1)	15 (39.4)	3.1	0.076
Anxiety and/or tension	23 (41.8)	16 (42.1)	0.001	0.970
Anger and/or irritability	36 (65.4)	16 (42.1)	4.97	0.026
Depressed or low mood	27 (49.1)	12 (31.5)	2.83	0.092
PMS^a^	31 (56.4)	12 (31.5)	5.55	0.018
PMDD^b^	12 (21.8)	5 (13.2)	1.12	0.280
No PMS or PMDD	12 (21.8)	21 (55.2)	10.98	0.001

^a^ PMS: Premenstrual syndrome

^b^ PMDD: Premenstrual dysphoric disorder

Forty five (27%) students with PMS or PMDD reported that they were not able to perform their prescheduled activities. Thirty of the 43 students in the case group (69%) and 14 of the 17 students in the control group (82%) who met the criteria for a diagnosis of PMS or PMDD used medications to alleviate their symptoms. However, 22 medical students out of 60 (36%) who met the criteria for PMS or PMDD did not use any medications.

## Discussion

Premenstrual tension was first defined in 1930s, when women entered the workforce.[8] It is stated that women who had never worked outside the home were less likely to report PMS.[9] Twenty four-hour shift work in internship programs involves a burden of responsibilities, physical activities and insomnia which seems to be able to serve as a potent stressor. This study shows that 24-hour shifts increases the rate of PMS in a sample of female medical school interns. In contrast, the difference in the frequency of PMDD between case and control groups was not statistically significant. Twenty two (36%) students with PMS or PMDD considered their symptoms to be normal for a menstrual cycle, supporting the idea that many women tolerate their symptoms believing it to be an unavoidable part of being female.[10]

Surveys of women of reproductive age reported a rate of 20% to 50% for PMS and 3% to 9% for PMDD.[1][11][12][13] However, the present study showed that 46.2% of the medical students suffered from PMS and 18.2% had PMDD which shows that medical students are a high risk group for PMS and PMDD. Decreased energy and irritability were the most frequent symptoms experienced in this study, and 27% of the women with PMS or PMDD were not able to attend their prescheduled programs confirming the statement that the burden of PMS and PMDD as measured by disability-adjusted life years is similar in magnitude to that associated with major recognized disorders.[1]

Although the prospective daily self-reporting forms used in this study could have minimized memory errors, we acknowledge the limitations of this study such as its small sample size, probability of presence of other life stressors, the absence of objective severity scales and the overlap between severe PMS and PMDD, which may have induced diagnostic errors.

The present data showed that work stress may exacerbate PMS. We believe that in spite of the high prevalence of PMS and PMDD, this condition is still under-recognized and undertreated. Using self-help approaches to induce self-awareness, psychological and psychiatric interventions, and appropriate medications may help susceptible women with high work stress to overcome this cyclic condition in order to increase their productivity and quality of life.
